# Presence of depression and anxiety before and after coronary artery bypass graft surgery and their relationship to age

**DOI:** 10.1186/1471-244X-7-47

**Published:** 2007-09-12

**Authors:** Jens-Holger A Krannich, Peter Weyers, Stefan Lueger, Michael Herzog, Thomas Bohrer, Olaf Elert

**Affiliations:** 1 Department of Cardio-Thoracic Surgery, University of Wuerzburg, Germany; 2 Department of Clinical Psychology, University of Wuerzburg, Germany; 3 Deegenbergklinik, Bad Kissingen, Germany; 4 Department of Otorhinolaryngology, Head and Neck Surgery, University of Greifswald, Germany

## Abstract

**Background:**

Scientific literature on depression and anxiety in patients with coronary heart disease (CHD) consistently reports data of elevated anxiety and depression scores indicating clinically relevant quantities of these psychopathological conditions. Depression is considered to be a risk factor for the development of CHD and deteriorates the outcome after cardiac rehabilitation efforts. The aim of our study was to evaluate the presence of clinically relevant anxiety and depression in patients before and after coronary artery bypass grafting (CABG). Additionally we evaluated their relationship to age because of the increasing number of elderly patients undergoing CABG surgery.

**Methods:**

One hundred and forty-two consecutive patients who underwent CABG in our hospital were asked to fill in the "Hospital Anxiety and Depression Scale – German Version (HADS)" to measure depression and anxiety scores two days before and ten days after CABG surgery. Differences between these pre- and post-surgical scores were then calculated as means for changes, and the amount of elevated scores were appraised. In order to investigate the relationship between age and anxiety and depression, respectively, Spearman correlations between age and the difference scores were calculated. In addition, ANOVA procedures with the factor "age group" and McNemar tests were calculated. Therefore the sample was divided into four equally sized age groups.

**Results:**

25.8% of the patients were clinically depressed before and 17.5% after surgery; 34.0% of the patients were clinically anxious before and 24.7% after surgery. This overall change is not significant. We found a significant negative correlation between age and the difference between the two time points for anxiety (Spearman rho = -.218; p = 0.03), but not for depression (Spearman rho = -.128; p = 0.21). ANOVA and McNemar-Tests revealed that anxiety scores and the number of patients high in anxiety declined statistically meaningful only in the youngest patient group. Such a relationship could not be found for depression.

**Conclusion:**

Our data show a relationship between age and anxiety. Younger patients are more anxious before CABG surgery than older ones and show a decline in symptoms while elderly patients show hardly any change.

## Background

The prevalence of depression in patients with diagnosed coronary heart disease (CHD) is quoted between 20 and 45% [1,2]. Elevated anxiety scores have been reported for 20 to 55% [3,4]. The same prevalence ratios have been found for patients undergoing CABG surgery [5].

Depression is regarded as an independent risk factor for arteriosclerotic deposits in coronary arteries [6]. The pathophysiological processes to explain this relationship are hypercortisolaemia related to e.g. insulin resistance, sympathetic vagal dysbalance related to e.g. disturbed regulation of blood pressure, and an unfavourable lifestyle like cigarette smoking [7]. Depression does not only account for a raise in first time CHD manifestation, but it is also related to the success of secondary and tertiary prevention:

1. presurgical [8] as well as postsurgical [9] depression increases physical and psychosocial morbidity six month [8] and five years [9] after CABG-surgery,

2. preoperative depression is a significant predictor of mortality in the period 30 days after CABG-surgery [10],

3. depressed patients have a lower functional status [11] and less health status benefits six month after CABG surgery [12],

4. post myocardial infarction (MI) depression reduces the probability of secondary CHD prevention four month after MI [13].

Depressed patients with MI are reported to increase economical burdens [14]. Based on a Canadian sample, patients being depressed during the first year following MI produce approximately 41% higher health-care costs than non-depressed patients [14].

In contrast to depression, anxiety is not considered an independent risk factor for MI. Depression is associated with higher postoperative mortality and morbidity. The same relationship was found for acute preoperative anxiety but not for trait anxiety [15]. It is reported, however, that the risk of sudden cardiac death is elevated by anxiety disorders. Pathophysiologically ventricular arrhythmias are considered to explain this phenomenon [16].

Patients who undergo CABG surgery nowadays are older than a decade before. In Germany, in the year 2004 42.8% of all procedures using cardiopulmonary bypass were performed in patients older than 70 years, whereas in 1994 the percentage was 24.9% [17]. Increased age is considered to be a high risk factor for perioperative mortality. Age is one of the few factors which is included in all of the formulas to calculate mortality risk [18].

Furthermore, it is known that depression affects the life of elderly people more than any physical disorder [19]. No consistent pattern across studies investigating age differences in the occurrence of anxiety and depression is available in the current literature [20]. To our knowledge, the prevalence of clinically relevant anxiety and depression in patients undergoing CABG-surgery in relation to age has not been investigated yet.

The objective of the present study was to evaluate the proportion of clinically relevant anxiety and depression and the number of symptoms for both conditions in CHD patients two days before and ten days after CABG surgery in relation to age.

## Methods

### Design

142 consecutive patients who underwent CABG surgery at our institution were included in the study. 70 patients were surveyed from January to June 2002 while 72 patients were examined from January to June 2003. They were also part of another investigation. Patients in 2003 were involved in a program to enhance motivation for a change in lifestyle (treatment) whereas patients in 2002 were not involved in this program. In the treatment group patients had the opportunity to take part in a special lecture and an individual lesson about how to prevent reocclusion of the bypass grafts whereas the usual care group was not provided with such information [21]. The data from 2002 and 2003 were combined since there were no significant differences in terms of depression and anxiety at both measurement points between the two groups.

The study was approved by the institutional review board of the Cardiothoracic Department of Wuerzburg University, and each patient gave written informed consent to participate.

All patients fulfilled the following criteria: native German speaker, ability to read and write, admission to hospital on a working day, no instable angina pectoris, no neurological deficits, no dementia, no emergency CABG surgery, and signed agreement to participate.

### Measurement

Anxiety and depression were measured with the "Hospital Anxiety and Depression Scale – German Version" (HADS) [22]. Patients were examined two days before and ten days after CABG surgery. The HADS is a short self report questionnaire to screen for anxiety and depression. It was especially developed for hospitalized persons with physical illness. The questionnaire consists of 14 items, seven to measure anxiety and seven to measure depression. The items are formulated as symptoms referring to the last seven days. Each statement can be answered by choosing one out of four options. The answers differ from question to question. There are two ways to analyse HADS values. First, the raw scores for depression and anxiety can be counted in order to measure the number of symptoms. Second, the raw scores can be used to classify patients with elevated and not elevated depression and anxiety scores, respectively. According to research literature [23,24] the cut-off for elevated anxiety and depression scores is = 8. Sensitivity and specifity for this cut-off are 0.70 and 0.90, respectively [23].

### Statistics

McNemar tests were used to find out if there are different proportions of patients with elevated anxiety and depression scores before and after CABG surgery. The raw scores were examined for differences with t-tests for dependent measurements. The relationships between age and anxiety and depression, respectively, were evaluated with nonparametric Spearman rho correlations between patients' age and the differences of the pre- and the post-surgical values in the HADS. In order to calculate these differences in anxiety and depression, the values ten days after CABG surgery were subtracted from the values two days before CABG surgery. A positive result indicates higher pre-surgical values, and a negative result indicates higher post-surgical values. A zero result indicates no change between the two measurement points.

In addition, ANOVA procedures for repeated measurements were conducted. The group variable was constructed by creating four equally sized age groups. For that purpose the patients were divided into four equally sized age groups by means of quartiles. The quartile ranges were 36 to 60 years (n = 36), 61 to 66 years (n = 36), 67 to 72 years (n = 35), and 73 to 78 years (n = 35). Thus, with ANOVA we could evaluate the main effects for the variables "age group" and "time" (repeated measurements) and the interaction effect of both. Given a significant effect, differences were tested with the post-hoc test "Fishers Least Significant Differences" test (LSD). Statistical analyses were conducted using "STATISTICA 7" (StatSoft Inc. Tulsa, OK, USA). A p value of .05 or less was considered to indicate statistical significance.

Missing data were not substituted because of the association between the numbers of missing values and the independent variable age (see result section). Therefore, data from 97 patients were analysed.

## Results

Ten patients (7.0%) suffered from medical complications so they could not fill in the HADS ten days after CABG surgery. A significant age dependent increase in medical complications could not be detected even though half of the patients who suffered from medical complications were older than 72 years (see Table 1). (Fisher's exact p = 0.24 for the overall test and p = 0.08 for comparing the youngest with the oldest patient group).

**Table 1:** Percentage of medical complication that detained patients from editing the HADS and proportion of HADS completed divided into age groups (N = 142)

Age group [years] (n)	Percentage of medical complications	Percentage of HADS filled in two days before CABG surgery	Percentage of HADS filled in ten days after CABG surgery
36–60 (36)	2.7%	100%	86.1%
61–66 (36)	5.5%	91.6%	80.5%
67–72 (35)	5.7%	94.3%	62.8%
73–78 (35)	14.3%	85.7%	45.7%
Total	7.0%	92.9%	69.0%

The percentage of patients who filled in the HADS was different between the age groups (see Table 1). Before surgery there were no significant differences (Fisher's exact: p = 0.12). Ten days after CABG surgery, however, we found highly significant differences (*Chi-square *= 16.7, *df *= 3; *p *< 0.001). The older the age group was the less patients filled in the HADS. Statistically significant differences resulted only by comparing age group "36–60 years" with age group "73–78 years" (*Chi-square *= 12.9; *df *= 1; *p *< 0.001) and age group "36–60 years" with age group "67–72 years" (*Chi-square *= 5.1; *df *= 1; *p *= 0.02).

Data analyses with the categorical classification indicate for anxiety that 34.0% of the patients before, 24.7% after CABG surgery, and 16.5% at both time points were highly anxious. 25.8% of the patients were depressed two days before CABG surgery, 17.5% ten days after, and 9.3% at both questionnings. For both, anxiety and depression, the changes in these ratios are not significant (McNemar test). However, by testing the accompanied continuous HADS scores a statistically significant decline could be found for anxiety (before surgery: *m *= 6.78; *SD *= 3.86, after surgery: *m *= 5.38; *SD *= 3.75, *T*(96) = 3.82; *p *< 0.001) as well as for depression (before surgery: *m *= 5.65; *SD *= 3.71, after surgery: *m *= 4.51; *SD *= 4.08, *T*(96) = 3.35; *p *= 0.001).

We could find a significant correlation between age and the changes in anxiety (Spearman rho = -.218; p = 0.03), but not between age and the changes in depression (Spearman rho = -.128; p = 0.21). The former indicates that the younger the patient is the larger is the difference between pre- and post-surgical anxiety scores. Specifically, younger patients show a stronger decline in anxiety. These results were confirmed for the continuous HADS anxiety scores by ANOVA procedures. We could prove a significant interaction between the factors "age group" and "time" for anxiety (*F*(3, 93) = 2.89; *p *= 0.03), and a significant effect for the factor "time" (*F*(1, 93) = 9.86; *p *= 0.002). The anxiety scores are significantly lower ten days after CABG-surgery than two days before surgery. However, and changes dependent on the age group, since the LSD post-hoc test revealed a significant decline in anxiety only in the youngest group (36–60 years) between the two measurement points (*p *< 0.001) (see table 2 for descriptive scores).

**Table 2:** Mean values and (SD) in anxiety and depression two days before and ten days after CABG surgery divided into four age groups

Age group [years]	Anxiety two days before CABG	Anxiety ten days after CABG	Depression two days before CABG	Depression ten days after CABG
36–60 (n = 31)	7.54 (4.41)	4.77 (3.68)	6.25 (4.31)	4.87 (4.17)
61–66 (n = 29)	6.72 (3.69)	5.48 (3.74)	5.21 (3.47)	4.93 (4.27)
67–72 (n = 21)	6.90 (3.40)	6.09 (3.75)	5.33 (3.95)	4.23 (3.13)
73–78 (n = 16)	5.25 (3.45)	5.43 (4.05)	5.75 (2.54)	5.37 (4.78)

The nonparametric McNemar test supports the post-hoc findings, because the amount of high scorers in anxiety is diminished only in the youngest patient group (*McNemar *= 4.16; *df *= 1; *p *= 0.04).

For depression we could only find a significant effect of the factor "time" (*F*(1, 93) = 9.85; *p *= 0.003). Depression scores are lower after the CABG-surgery. The number of high scorers does not change over time. (see Table 3 for the numbers of high scorers).

**Table 3:** Number and percentages [%] of high-scorers in anxiety and depression (HADS value ≥ 8) two days before and ten days after CABG surgery divided into four age groups

Age group [years]	Anxiety high-scorers two days before CABG	Anxiety high-scorers ten days after CABG	Depression high-scorers two days before CABG	Depression high-scorers ten days after CABG
36–60 (n = 31)	11 (35.5)	5 (16.1)	9 (29,0)	3 (9.7)
61–66 (n = 29)	9 (31.0)	6 (20.7)	6 (24,1)	7 (20.7)
67–72 (n = 21)	9 (42.9)	7 (33.3)	5 (23.8)	3 (14.3)
73–78 (n = 16)	4 (25.0)	6 (37.5)	5 (31.2)	4 (25.0)

## Discussion

Before CABG surgery, age did not seem to have any influence on the rate of completion of the HADS. After surgery, however, completion rate decreased significantly with age. This cannot be explained by higher medical complication rates that prevented the patients from filling in the questionnaire. Potential explanations could be physical impairment, a general weakness, and/or the unfamiliar cognitive demand of the test putting too much strain on older patients. In addition, the questionnaire explicitly asks for mental concerns. According to our experience, older people are prone to react to questions like these with avoidance tendencies which could mask psychiatric comorbidity. One of our first assumptions was that patients with elevated anxiety or depression scores two days before CABG surgery would not fill in the HADS for a second time. However, this could not be supported by our data because the number of missing data ten days after CABG was independent of the status of anxiety and depression, respectively (i.e. high-scorers vs. normal scorers). The quantity of missing data is the main limitation of our investigation. However, the results are congruent with our clinical impressions.

The increased anxiety and depression rates in our study, compared to the general population, are in accordance with those described in the literature [1,5,8]. The decline in anxiety and depression scores from pre to post CABG surgery points to the fact that the patients are under considerable psychic strain before CABG surgery.

Furthermore, we could demonstrate negative correlations between age and changes in depression and anxiety scores. A closer inspection of these negative correlations and the ANOVA results revealed that the younger the patients are the larger is the decrease from pre- to post-surgery scores. There are some possible reasons for the specific age related pattern of anxiety and depression. Information about the surgery, which is routinely performed prior to surgery, may induce thoughts of one's own potential death, since death is mentioned as a possible complication of CABG surgery. These thoughts could have been present in the younger as well as in the older patients. It seems that older patients consider anticipated relief more than the strain imposed by angina pectoris. Younger patients, however, suffer from less physical limitations caused by CHD than the older ones. Therefore it is possible that before CABG surgery the younger patients are more affected by a potential deadly outcome than by the removal of physical CHD symptoms. Another possible explanation is that during their lives older patients were more frequently confronted with thoughts of their own death. As a consequence of knowing the stimulus "own death" patients may habituate resulting in less anxiety. In addition, younger patients may estimate their mortality risk and how long they will live. So, they might calculate a much greater loss than older patients, and stronger anxiety is elicited with the assumed number of years ahead of them. While discussing our results with a 75 year old male patient he mentioned another possible reason. Elder patients mostly have CHD for several years and may have become trained in getting into the hospital. Therefore, they are confronted with fewer new stimuli as compared to most of the younger patients.

Remarkably, the ratio of patients with elevated depression values at both time points is only 9.3%. Such a low ratio has not been reported before and may depend on the longitudinal design of our study, while other studies evaluate depression only at a single time point, e.g. before or shortly after CABG surgery.

## Conclusion

Based on the results of our study one may conclude: Many patients have noticeable mental distress before CABG surgery which has to be recognized and treated. The younger the patients are the higher is the decline of symptoms. The older the patient are the less change of symptoms is observed. According to these findings anxiety specific symptoms, e.g. tachycardia, should be kept in mind especially for younger patients before CABG surgery. In short term care and before surgery, treatment should predominantly focus on anxiety. Anxiolytic drugs should be prescribed as soon as possible, and potential threats should be avoided. In this regard, a trustful physician-patient-relationship is of importance. On the other hand, to sustain a good long-term outcome after CABG surgery treatment of depression surely is more important than treatment of anxiety. With some exceptions [25], for many patients the best depression therapy is a combination of antidepressants and psychotherapy. The basics of sufficient treatment, however, are reliable and valid diagnostic investigations.

## Competing interests

The authors declare that they have no competing interests.

## Authors' contributions

The authors designed the study and interpreted the results together. J-HK was responsible for patient acquisition, data analysis, and drafted the first version of the manuscript. PW, SL, MH, TB, OE contributed to data analysis and revised the manuscript critically. All authors have read and approved the final manuscript.

## Pre-publication history

The pre-publication history for this paper can be accessed here:


